# AI-driven multi-omics profiling of sepsis immunity in the digestive system

**DOI:** 10.3389/fimmu.2025.1590526

**Published:** 2025-05-20

**Authors:** Yuan Gao, Hong Chen, Ruolan Wu, ZuJun Zhou

**Affiliations:** Department of Emergency Medicine, Chonggang General Hospital, Chongqing, China

**Keywords:** sepsis, multi-omics, AI, immune response, biomarkers, gut microbiota

## Abstract

Sepsis is a life-threatening systemic inflammatory syndrome characterized by a complex immune biphasic imbalance. Monitoring of immune status has not yet been implemented in clinical practice due to lack of direct therapeutic utility. Immune dysregulation in sepsis patients is heterogeneous and dynamic. The use of artificial intelligence to drive the integration of multi-omics data, including genomics, transcriptomics, proteomics, and metabolomics, enables biomarker monitoring and immunoassays. This review revisits gut microbes as critical illness drivers and important regulatory players in sepsis immunity. It focuses on the synthesis of clinical biomarkers of sepsis and parameters related to the gut microenvironment with the help of artificial intelligence, enabling marker identification, immunostratification and predictive modeling. This feasible clinical decision-making algorithm based on “combinatorial typing” is an important tool for realizing precision medicine for sepsis patients.

## 1 Introduction

Sepsis is a dysregulated systemic immune response of the host to infection, often accompanied by severe systemic inflammation and multiple organ dysfunction syndrome (MODS), and is one of the leading causes of death in intensive care unit (ICU) patients. The Global Burden of Disease (GBD) study estimated that there were approximately 11 million sepsis-related deaths worldwide in 2017, accounting for nearly 20% of global deaths (1–4). Despite significant advances in antimicrobial therapy and critical care medicine over the past decades, sepsis deaths remain high, especially when early diagnosis is not timely or treatment strategies are inadequate (5, 6).

The complex pathomechanism of sepsis involves a biphasic imbalance characterizing the host immune response: i.e., early immune hyperactivation (manifested as systemic inflammatory response syndrome, SIRS) and late immune suppression (e.g., T-cell failure and immune tolerance), which predisposes patients to secondary infections and significantly affects long-term prognosis (7–10). Against the backdrop of the 2025 Japan influenza outbreak, dominated by the A(H1N1)pdm09 subtype, immunotargeted treatment strategies for severe cases have further highlighted the potential of immune system-based therapies in sepsis treatment (11–13). Additionally, the gut microbiota (gut microbiota) plays an important role in the regulation of immune homeostasis sepsis in of intestinal origin sepsis (14). novel immunomodulatory therapeutic strategies centered on restoration of intestinal microbiological homeostasis have gradually gained attention to assist in remodeling the host immune homeostasis, identify individual patient’s immune status and optimize early diagnosis, thereby improving survival and treatment outcome of septicemia patients.

Current conventional biomarkers used clinically for the diagnosis and evaluation of sepsis include procalcitoninogen (PCT), C-reactive protein (CRP), white blood cell count (WBC), and a variety of inflammatory cytokines (15–17).However, these conventional markers are often abnormally expressed in the presence of trauma, surgery, and non-infectious diseases, resulting in insufficient specificity and limited sensitivity in distinguishing sepsis from non-infectious inflammation, these traditional markers. In addition, due to the high degree of heterogeneity of immune imbalances in sepsis, it is difficult for a single biomarker to comprehensively reflect the complexity of the immune dynamics. Such limitations have prompted researchers to turn to more comprehensive and precise biomarker discovery methods to improve the accuracy of disease diagnosis and prognosis (18).

In recent years, with the rapid development of multi-omics technologies (including genomics, transcriptomics, proteomics, and metabolomics), the emergence of large-scale and high-dimensional biological data has posed a great challenge to the traditional means of analysis (19–22). Artificial Intelligence (AI), especially machine learning (ML) and deep learning (DL) algorithms are becoming an important tool in sepsis research due to their powerful data mining capabilities (23). AI algorithms are not only capable of efficiently integrating clinical data (e.g., electronic health records, physiological monitoring data) with multi-omics data, but also identifying complex non-linear features that cannot be captured by traditional methods, thus enabling more accurate patient stratification, immune status assessment and disease progression prediction (24–27).

The aim of this review is to explore the application of artificial intelligence (AI) and multi-omics technologies in the study of immune response in sepsis, focusing on the analysis of how AI integrates genomics, transcriptomics, proteomics and metabolomics data for the discovery of new and efficient biomarkers to optimize the early diagnosis, risk assessment, and individualized therapeutic strategies for sepsis. In addition, we will also discuss the challenges and future research directions of AI and multi-omics in the process of clinical translation, with the aim of providing theoretical basis and practical reference for the promotion of sepsis precision medicine.

## 2 Applying AI and machine learning to the immune response in sepsis

AI has evolved from its early days, limited by reliance on expert rules, to its current incarnations of ML and DL, both of which are subdivided into Supervised Learning and Reinforcement Learning. Both are subdivided into supervised, unsupervised and reinforcement learning. ML involves algorithms that define their own rules from input data through iterative training and refinement, without human intervention (28). DL uses multi-layer neural networks to simulate the way the human brain processes information, such as the large language models that are currently popular (ChatGPT, Deepseek) (29, 30). Due to its complex structure and large computational volume, it requires more high-performance computing resources than traditional machine learning methods. And because its internal working mechanism is complex and difficult to be understood by human beings (i.e., the “black box” effect), how to explain its decision-making process and how to be accountable when something goes wrong is an important challenge at present (31).

Early recognition of sepsis can reduce mortality, and current common clinical surveillance relies on predefined rules (e.g., specific symptoms or signs). In the quest for more effective disease management strategies, the development of Machine Learning Algorithms (MLA) for sepsis prediction algorithms is more dynamic and time-sensitive (32–34). Moreover, machine learning techniques effectively mine patterns in histological data to help classify and identify different biometric subgroups of patients, revealing the heterogeneity of sepsis.

Neonatal sepsis as a cause of high infant mortality rate (35). Due to the rapid progression of symptoms, timely diagnosis of neonatal sepsis requires a combination of clinical indicators, inflammatory biomarkers, and blood cultures to reduce the risk of mortality (36). These diagnostic complexities can be leveraged with machine learning models to process large, structurally complex datasets, enabling stratified, individualized prediction and early warning.

## 3 Intestinal immune dysregulation in sepsis

The gastrointestinal tract is integral to the pathophysiologic process of sepsis and plays a catalytic role in driving and sustaining multi-organ dysfunction (37–39). The establishment of intestinal immunity is dependent on both the intestinal flora and the physical barrier between the host and microorganisms and the immune cells at the barrier (14, 40).

The mucosal immune system consists of GALT (gut-associated lymphoid tissue, including Peyer’s patches and isolated lymphoid follicles) at the induction site and the lamina propria and intestinal epithelium at the primary effector site (41–43). As the outermost outpost, the intestinal epithelium is the active component of immunity and promotes the innate immune response (44, 45). The intestinal epithelium is divided into absorptive and lymphoid-associated epithelia (46). T cells, B cells, and dendritic cells accumulate in the subepithelial dome region of lymphoid tissue, creating a protective immune barrier (47). Dependent on M-cell (Peyer’s patches located in the intestinal epithelium) transport, macromolecules and Ag from the lumen are sampled and presented locally to T cells (48). Whereas the lamina propria is enriched with effector T cells (Th1, Th17), regulatory T cells (Treg), plasma cells (secreting IgA), and tolerogenic CX3CR1+ macrophages (Macs) (49, 50).

In the early stages of sepsis of intestinal origin, overwhelming infection induces a hyperinflammatory state in the organism, which leads to intestinal hyperpermeability. The compromised mucosal barrier permits the transfer of bacteria and their products (e.g., LPS, peptidoglycan, lipophosphatidic acid, etc.) to the mesenteric lymph nodes, liver, spleen, and bloodstream, activating the host immune system (51). The immune response is initially activated by the perception of pathogen-associated molecular patterns (PAMPs) by pattern recognition receptors (PRRs), which in turn induce a wide range of biological responses (52, 53).

In the post-acute phase of sepsis, the immune response enters a state of hyporesponsiveness. When the phase of immune paralysis (i.e., immune imbalance) takes center stage, it is characterized by hypofunction of antigen-presenting cells (APCs), depletion of T-cells, and reprogramming of antigen-presenting cells (54, 55). These cells have a reduced ability to produce pro-inflammatory cytokines in response to stimulation, making the organism highly susceptible to secondary infections. At the same time, there is an increase in death-associated molecules (programmed cell death-1 (PD-1), caspases) and a decrease in the expression of HLA-DR (54, 56, 57). A decrease in commensal bacteria and colonization by conditionally pathogenic bacteria (e.g., Enterobacteriaceae, Clostridium difficile) during periods of high inflammation and an imbalance in the intestinal microecology lead to an increased risk of secondary infections (58). This in turn drives sepsis and perpetuates the inflammatory response, inducing SIRS and increasing the prognostic burden of sepsis. It is shown that the microbiota is associated with systemic tolerance of the host immune system.

However, data suggest that not all T-cell responses are suppressed in sepsis survivors and that some specific CD4 T cells may be restored (59–61). For example, in microbial sepsis induced by cecum ligation and puncture (CLP), the influenza A virus (IAV) NP311-specific CD4 T cell population, CD4 T cells, showed Ag-dependent proliferation (62).

In conclusion, the pathophysiology of sepsis centers around a hyperinflammatory phenotype or an immunosuppressive phenotype. The high mortality rate in sepsis remains severe, mainly due to the lack of effective treatment strategies that can support the recovery of immune system function and reverse immune imbalances. Therefore, immunotherapy to target this dysregulation is expected to identify appropriate treatments and therapeutic windows.

## 4 Exploring novel biomarkers for sepsis based on multi-omics analysis

### 4.1 Genomics

**Figure 1:**
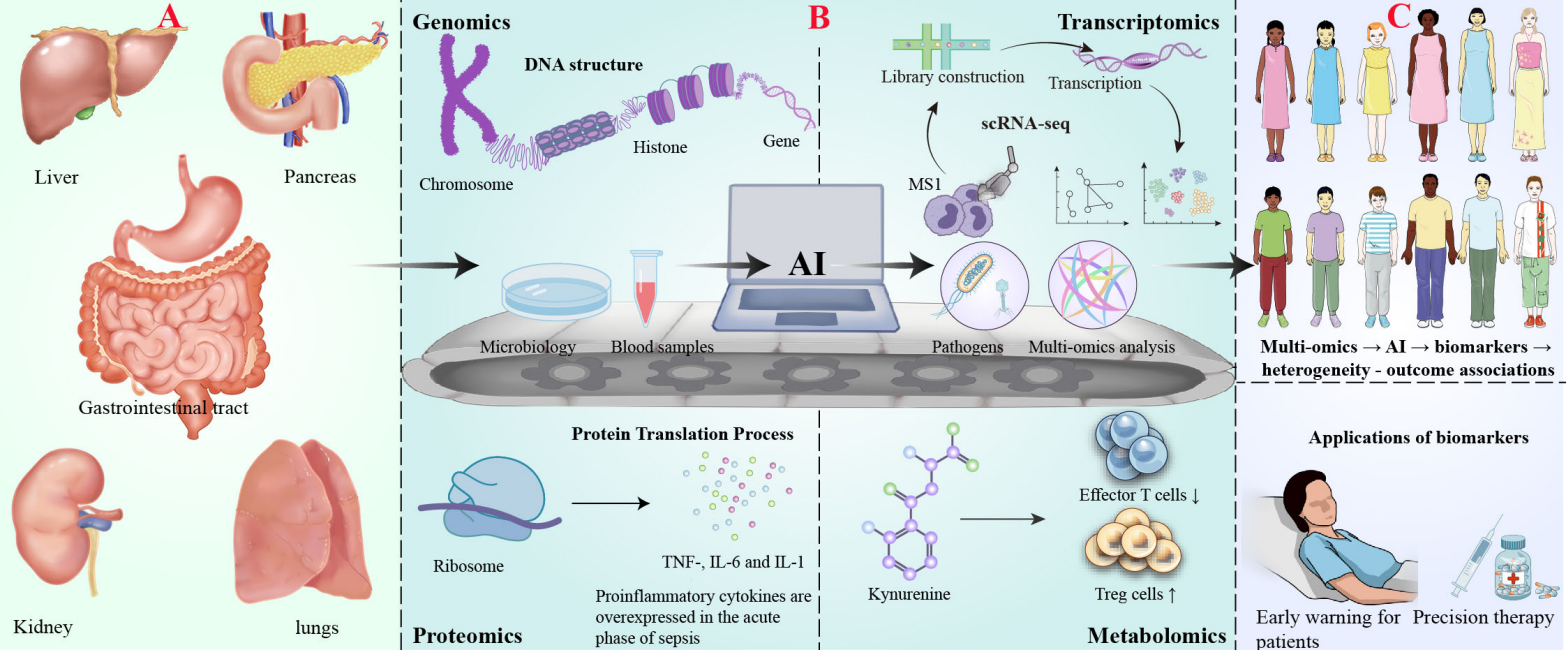
AI-driven multi-omics profiling for biomarker discovery in digestive system-associated sepsis. **(A)** Digestive infection and onset of sepsis. **(B)** AI-driven multi-omics analysis workflow: Demonstrates the analytical process of multi-omics technologies: genomics (top left, DNA structure), transcriptomics (top right, single-cell RNA sequencing technology), proteomics (bottom left, protein transcription process and representative elevated cytokines in sepsis) and metabolomics (bottom right, effect of ureide metabolic pathways on T cell function). The centrally located horizontal conveyor belt symbolizes the AI-driven data analysis process, including sample collection (petri dishes, patient blood), AI computational analysis (represented by a computer) and output of the analysis results (multi-omics data with pathogenic bacteria icon representing the identified markers). **(C)** Biomarker discovery and clinical application.

Through genome-wide association studies (GWAS) and analysis of single nucleotide polymorphisms (SNPs), the study revealed multiple genetic variants associated with immune response (63) (**Figure 1**). AI was able to mine genetic markers associated with susceptibility to sepsis and immune response from these genomic data (64, 65). For example, an integrative analysis based on publicly available datasets found that immune-related genes such as LTB4R, HLA-DMB, and IL4R were strongly associated with 28-day mortality in patients with sepsis, potentially influencing prognosis by modulating the strength of the immune response (66). In addition, differential expression analysis for MS1 cells from ICU-SEP and ICU-NoSEP patients revealed that the expression of PLAC8 gene was associated with sepsis, while CLU was specifically up-regulated in MS1 cells, which may be a new sepsis biomarker (67). AI analyzed these variations by deep learning algorithms, which could accurately identify the genes associated with immune response, for providing strong support personalized treatment of sepsis. For example, Sweeney et al. integrated transcriptomic data from 21 sepsis cohorts (1113 patients in total) and constructed four sepsis prognostic prediction models using supervised learning algorithms such as Gradient Boosting Trees. The study started with data downscaling and feature selection using batch effect correction and principal component analysis (PCA), followed by model training and evaluation in the training and validation sets. The final model obtained an area under the ROC curve (AUROC) of approximately 0.85 in the independent validation set, which significantly outperformed traditional clinical scoring systems (e.g., SOFA score). Through this AI-driven multi-omics data analysis process, the clinic is able to more accurately type and prognostically assess the patient’s immune status for more individualised diagnostic and therapeutic decisions [PMID: 36470834].

### 4.2 Transcriptomics

Transcriptomics reveals specific gene expression patterns associated with viral or bacterial infections by analyzing mRNA expression profiles in the blood and tissues of sepsis patients, for of sepsis providing new biomarkers personalized treatment (68, 69).

Heterogeneity of the immune system in sepsis patients could be identified by analysis of single-cell RNA sequencing (scRNA-seq) technology, which revealed significant changes in certain immune cell populations (70, 71). For example, the CD14+ monocyte subset (MS1) undergoes an expansion in sepsis patients and correlates with immunosuppressive states (72). Such techniques allow researchers to reveal the profound effects of sepsis on the immune system at the single-cell level, providing possible directions for future immunomodulatory therapies and precision medicine.

Whole blood gene expression profiling further revealed that different subtypes (immune response endotypes) of sepsis could be recognized. For example, the Mars1 endotype is characterized by an impaired immune response, mainly manifested by reduced expression of genes related to lymphocyte signaling and antigen presentation pathways, which is strongly associated with poor patient prognosis (73). In addition, similar immunosuppressive features are also seen in childhood sepsis in the A endotype (74, 75). The identification of these endotypes provides a basis for early stratification of patients with sepsis and promotes the development of personalized treatment strategies.

In studies of sepsis-associated liver failure (ACLF), activation of the transcription factor C/EBPβ has been found to be one of the key factors contributing to the loss of liver function. Studies have shown that C/EBPβ expression is significantly increased in both mouse models and human ACLF patients.C/EBPβ contributes to sepsis-induced liver failure by activating endothelial dysfunction and regulating Angiopoietin-1/Angiopoietin-2 ratio and HGF production (76).

These transcriptomic markers provide new molecular mechanisms for our understanding of sepsis-induced multi-organ failure, as well as for using new ideas failure. These markers as potential therapeutic targets to ameliorate sepsis-associated organ.

### 4.3 Proteomics

In the immune response to sepsis, proteomic studies have revealed the multiple roles of cytokines, chemokines, and other related proteins in immune regulation (77, 78). These proteins not only directly reflect the degree of activity of the immune system, but also play key roles in the transformation of an over-excited or immunosuppressed state.

Pro-inflammatory cytokines such as tumor necrosis factor alpha (TNF-α), interleukin 6 (IL-6), and interleukin 1 (IL-1) are overexpressed during the acute phase of sepsis, leading to immune over-responses, which in turn induces a SIRS and multi-organ failure (79–82). IL-10 may contribute to immune tolerance or inhibit immune responses and under specific circumstances increase the risk of secondary infections, but its effects are spatiotemporally specific (83, 84).

S100A8 and S100A9 are key calcium-binding proteins in sepsis, and their immunomodulatory effects are not limited to the systemic immune response, but also the intestinal immune system play an important role in (85). studies have shown that S100A8 and S100A9 are involved in intestinal inflammatory responses and immune cell recruitment. In patients with sepsis, the expression of S100A8 and S100A9 was significantly elevated at sites of inflammation such as the intestine and lungs, exacerbating the local immune response. Mice deficient in these proteins exhibited impaired immune function and an increased risk of bacterial spread outside the gut. Supplementation with S100A8 and S100A9 restored immune cell function and improved gut microbiota balance, thereby reducing the incidence of sepsis (86).

These proteins not only serve as immune markers, but may also be novel targets for regulating intestinal immune responses, providing new ideas for personalized treatment of sepsis.

### 4.4 Metabolomics

Metabolomics is the study of small molecule metabolites and their changes in living organisms, and in recent years, metabolomics has played an important role in the study of immune response in sepsis (87, 88). Immune response is closely related to metabolism, and the activation and functional demands of immune cells are often accompanied by alterations in metabolic pathways, and metabolites, in turn, modulate the metabolic pathways, and the strength and direction of immune responses.

During the immune response to sepsis, significant changes in metabolic pathways occur. For example, sepsis-induced abnormalities in amino acid metabolism are closely associated with immunosuppression. It has been found that tryptophan, when metabolized to kynurenine via the indoleamine-2,3-dioxygenase (IDO) pathway, inhibits T-cell proliferation, promotes T-cell apoptosis, and enhances T-regulatory cell (Treg) production, leading to the development of an immune tolerance state (89).

Furthermore, in sepsis, dysregulation of glycolysis is closely linked to functional alterations in immune cells. It was found that in immune-mediated diseases such as acute liver injury, protein 4.1R promotes the polarization of M1-type macrophages by regulating glycolytic metabolism, driving immune activation and thus exacerbating inflammatory responses (90).

These metabolites, as potential prognostic markers, need to be further clinically validated and standardized for application in practical diagnosis and treatment. In addition, the combination of metabolomics and genomics, especially through multimodal genomics techniques, can reveal systemic biological changes in sepsis and provide a basis for the discovery of new therapeutic targets.

The immune response to sepsis is complex and diverse, and with the advancement of multi-omics technology, AI technology can effectively integrate big data from genomics, transcriptomics, proteomics and metabolomics to reveal potential biomarkers. These markers are important for early diagnosis of sepsis, assessment of immune response, and personalized treatment.

## 5 Discussion

Accurate analysis of dysregulated immune responses and the intestinal microenvironment is critical for the next step in optimizing the treatment of sepsis patients or improving the prognosis of critically ill patients. In immunomodulatory therapy research, specific biomarkers show potential in screening patient populations. Oral nanomedicinal immunotherapy has demonstrated unique advantages in modulating sepsis-associated gastrointestinal tract (GIT) factors with a strategy to restore a healthy gut microbiome: 1) better targeting of the GIT; 2) convenience of self-administration; and 3) better patient compliance (91).

Currently, stratification efforts have been combined with immunologic analyses, and the application of high-dimensional histology techniques has opened up new opportunities for clinical and translational sepsis research. Compared with traditional single-cell genomics, spatial genomics is able to simultaneously preserve the gene expression information of cells and their spatial localization in tissues, revealing the dynamics of immune cells in the pathological environment (92–94). Spatialomics can be used in sepsis research to resolve the spatial heterogeneity of the inflammatory microenvironment and reveal the distribution of immune cells in the gut, liver, lungs and other organs. Combined with AI techniques (e.g., deep learning and graph neural networks), it can optimize data integration, predict changes in immune cell dynamics, and identify key biomarkers. Although the technology has been applied to precision oncology, clinical translation in sepsis still faces challenges such as data standardization and experimental design optimization (95). In the future, AI-driven spatial genomics is expected to drive the development of precision immunomodulation and personalized therapy.

Dysbiosis of the gut microbiota may increase the risk of sepsis and its triggered organ dysfunction. Although sepsis is uncommon in the context of leaky gut barrier, the risk of bacterial translocation remains, especially in specific clinical situations such as neutropenia. This does not mean, however, that studying the gut (especially the microbiome) is not revealing for the treatment of critically ill septic patients. A significant reduction in the number of butyrate-producing bacterial species is observed in critical illness, which is associated with epithelial cell loss, reduced mucosal tolerance, and bacterial translocation (96). This suggests that balancing the host immune response by modifying the gut microbiome before or during a sepsis episode may reduce sepsis morbidity and mortality.
